# Pulmonary Abnormalities in Mice with Paracoccidioidomycosis: A Sequential Study Comparing High Resolution Computed Tomography and Pathologic Findings

**DOI:** 10.1371/journal.pntd.0000726

**Published:** 2010-06-29

**Authors:** Damaris Lopera, Tonny Naranjo, José Miguel Hidalgo, Bernardo Miguel de Oliveira Pascarelli, Jairo Hernando Patiño, Henrique Leonel Lenzi, Angela Restrepo, Luz Elena Cano

**Affiliations:** 1 Medical and Experimental Mycology Group, Corporación para Investigaciones Biológicas, Medellín, Colombia; 2 School of Health Sciences, Universidad Pontificia Bolivarina, Medellín, Colombia; 3 Radiology Department, Hospital Universitario San Vicente de Paúl, Medellín, Colombia; 4 Laboratory of Pathology, Instituto Oswaldo Cruz, Fundação Oswaldo Cruz, Rio de Janeiro, Brasil; 5 Medical School, Universidad de Antioquia, Medellín, Colombia; 6 Radiology Department, Clínica las Américas, Medellín, Colombia; 7 Microbiology School, Universidad de Antioquia, Medellín, Colombia; René Rachou Research Center, Brazil

## Abstract

**Background:**

Human paracoccidioidomycosis (PCM) is an endemic fungal disease of pulmonary origin. Follow-up of pulmonary lesions by image studies in an experimental model of PCM has not been previously attempted. This study focuses on defining patterns, topography and intensity of lung lesions in experimentally infected PCM mice by means of a comparative analysis between High Resolution Computed Tomography (HRCT) and histopathologic parameters.

**Methodology:**

Male BALB/c mice were intranasally inoculated with 3×10^6^ *Paracoccidioides brasiliensis* (*Pb*) conidia (*n = 50*) or PBS (*n = 50*). HRCT was done every four weeks to determine pulmonary lesions, quantify lung density, reconstruct and quantify lung air structure. Lungs were also analyzed by histopathology and histomorphometry.

**Results:**

Three different patterns of lesions were evidenced by HRCT and histopathology, as follows: nodular-diffuse, confluent and pseudo-tumoral. The lesions were mainly located around the hilus and affected more frequently the left lung. At the 4^th^ week post-challenge HRCT showed that 80% of the *Pb*-infected mice had peri-bronchial consolidations associated with a significant increase in upper lung density when compared with controls, (−263±25 vs. −422±10 HU, *p<0.001*). After the 8^th^ and 12^th^ weeks, consolidation had progressed involving also the middle regions. Histopathology revealed that consolidation as assessed by HRCT was equivalent histologically to a confluent granulomatous reaction, while nodules corresponded to individual compact granulomas. At the 16^th^ week of infection, confluent granulomas formed pseudotumoral masses that obstructed large bronchi. Discrete focal fibrosis was visible gradually around granulomas, but this finding was only evident by histopathology.

**Conclusions/Significance:**

This study demonstrated that conventional HRCT is a useful tool for evaluation and quantification of pulmonary damage occurring in experimental mouse PCM. The experimental design used decreases the need to sacrifice a large number of animals, and serves to monitor treatment efficacy by means of a more rational approach to the study of human lung disease.

## Author Summary

Paracoccidioidomycosis (PCM) is a fungal infection caused by the dimorphic fungus *Paracoccidioides brasiliensis.* It occurs preferentially in rural workers in whom the disease is severe and may cause incapacitating pulmonary sequelae. Assessment of disease progression and treatment outcome normally includes chest x-rays or CT studies. Existing experimental PCM models have focused on several aspects, but none has done a radiologic or image follow-up evaluation of pulmonary lesions considered as the fungus primary target. In this study, the lungs of mice infected with fungal conidia were studied sequentially during the chronic stage of their experimental mycosis by noninvasive high resolution medical computed tomography, and at time of sacrifice, also by histopathology to characterize pulmonary abnormalities. Three basic lung lesion patterns were revealed by both techniques: nodular-diffuse, confluent and pseudo-tumoral which were located mainly around the hilus thus accurately reflecting the situation in human patients. The experimental design of this study decreases the need to sacrifice a large number of animals, and serves to monitor treatment efficacy by means of a more rational approach to the study of human pulmonary diseases. The findings we are reporting open new avenues for experimental research, increase our understanding of the mycosis pathogenesis and consequently have repercussions in patients' care.

## Introduction

Human paracoccidioidomycosis (PCM) is an endemic fungal infection of pulmonary origin that disseminates to different sites, notably oral mucous membranes, skin, adrenal glands and reticuloendothelial system. The disease tends to run a chronic progressive course while acute cases are more unusual. This mycosis is caused by *Paracoccidioides brasiliensis*, a thermally dimorphic fungus [1].

Primary infection in humans occurs in the lungs, where it causes chronic granulomatous inflammation of the parenchyma leading to fibrosis and severe restriction of respiratory function [2].

We have developed a model of pulmonary PCM in male BALB/c mice induced by the intranasal inoculation of *P. brasiliensis* conidia [3]. This model allowed to evaluate histopathologically and immunologically the pulmonary tissue responses occurring during the active and residual stages of the processes [4], [5], [6]; however, radiological follow-up evaluation of pulmonary lesions in the experimental model of PCM have not been previously described.

Non-invasive radiological imaging has recently gained considerable interest in basic and preclinical research for monitoring disease progression and assessing therapeutic efficacy [7]. One of the most noteworthy attributes of non-invasive imaging is the ability to obtain data from individual animals at multiple time points. Therefore, the number of animals required for a study can be minimized [8]. Additionally, each pixel in the image has a value that can be mapped to the density of the tissue being imaged [9]. Furthermore, neither conventional histological analysis nor pulmonary function test provide information, in alive animals, on the three-dimensional (3-D) distribution of lesions over the entire lung volume [10].

Recent improvements in spatial resolution capacity have made possible to manufacture scanners specifically designed for imaging small animals (microscopic computed tomography - “micro-CT”), which produces images with spatial resolution of 50–100 µm [11]. However, the radiation dose delivered to the animal during micro-CT imaging may approach 5% of the median lethal dose in mice (LD50), potentially limiting the number of repeated studies that could be performed over time [12]. Furthermore, radiation exposure from repeated micro-CT scans may have an effect on skeletal growth in normal animals [13].

Some studies reported clinical scanners designed primarily for human application but employed, nonetheless, to follow-up lung fibrosis and tumor progression in mice [14], [15], [16]. However, only few studies have described and followed-up experimental mycoses using computed tomography (CT) [17] and no studies have been published using this radiological tool in experimental pulmonary PCM.

Recognition and monitoring of CT patterns associated with this model of disease could improve our understanding of anatomo-spatial distribution of lesions and their time course in the same animal. Also, it appears possible to identify differences between manifestations in human and experimental PCM models and, finally, imaging could also be used to evaluate *in vivo* therapeutic responses.

This study focus on a comparative analysis between high-resolution computed tomography (HRCT) and histopathological parameters, determining usefulness of performing noninvasive conventional medical X-ray tomography in the follow up of sequential lung lesions in the experimental PCM model induced in mice by conidial inoculation.

## Materials and Methods

### Ethics Statement

All animals were handled according to the national and international guidelines for animal research and experimental protocols were approved by Corporación para Investigaciones Biológicas (CIB) research ethics committee.

### Animals

BALB/c mice were originally obtained from Taconic Farms, Inc. Quality Laboratory Animals and Services for Research, New York, USA with the breeding colony being then expanded at the Corporación para Investigaciones Biológicas (CIB), Medellin, Colombia. Male mice, 7 weeks old and approximately 20 g in weight were used in this study. Mice were divided into 2 groups: non-infected control mice (*n = 50*) and *P. brasiliensis* infected mice (*n = 50*).

### Fungus

A *P. brasiliensis* strain registered at the American Type Culture Collection (Rockville, MD), ATCC-60855, was used in all experiments. This strain was originally isolated from a Colombian patient and it is known to produce abundant conidia (natural infectious propagules) [18], and cause a progressive chronic disease [19]. The strain was previously passed though mice to restore virulence and then used for production of conidia. The fungus was maintained at 18°C in its mycelial form by successive transfers on the modified synthetic McVeigh and Morton (SMVM) medium [20]. The growth was then transferred to an Erlenmeyer flask with liquid SMVM and incubated for 10–15 days (18°C) with constant shaking at 150 rpm (Model G-2 gyratory shaker, New Brunswick Scientific, Co. New Brunswick, N.J.). After this time, growth was collected, homogenized in a blender (Eberbach container assembly semi-micro press with fit cover) for 15–20 seconds in four intervals of four seconds each, and plated in Petri dishes containing a media that stimulates conidia production, namely, water agar medium and dextrose salts agar [21]. Culture dishes were washed with 0.85% saline solution plus 0.01% Tween-20; this suspension was then shaken at 250 rpm for 45 min at 18°C in an Erlenmeyer flask containing glass beads. The homogenized suspension was sonicated twice for 15 seconds at 7 Hz at 4°C with one minute intervals (Sonicator model 200, Branson Ultrasonic Co, Danbury,CT). The fungal slurry was then poured through a sterile syringe packed with glass wool. The conidia suspension that passed through the glass wool was concentrated by centrifugation. The number and viability of the conidia were determined by the fluorescein diacetate-ethidium bromide fluorescence method [22]. The viability of the conidia was consistently higher than 90% of the total number of conidia counted. The inoculum was then adjusted so that 0.06 ml contained approximately 3×10^6^ viable conidia [4].

### Experimental Infection

Mice were anesthetized by the intramuscular injection of a solution containing Ketamin hydrochloride (Park, Davis & Company, Berlin, Germany; 100 mg/kg) and Xylazine (Bayer, Brazil 10 mg/kg) [23]. When deep anesthesia was obtained, 3×10^6^ conidia (in 0.06 ml of the inoculum) were instilled intranasally. Control mice received an intranasal inoculum of 0.06 ml of saline [4].

### High-Resolution CT

**Figure 1:**
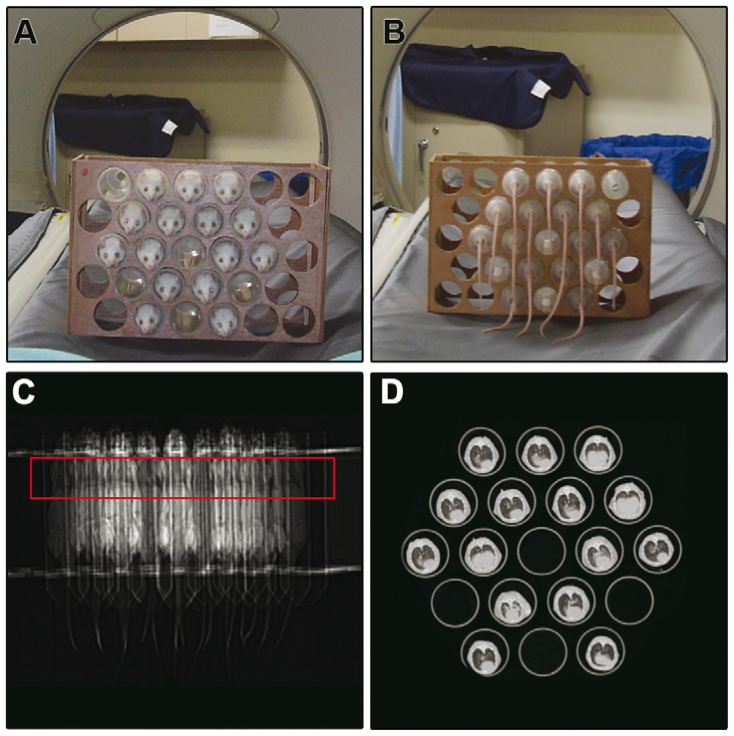
Arrangement of multiple mice for high resolution CT of lungs using a clinical CT-scanner. (A) Animal wood box holder with multiple anesthetized mice placed in prone position inside polypropylene tubes (anterior view). (B) Posterior view of the same structure. (C) Scout view or scanogram to plot the chest area where slice images are obtained (area labeled in red). (D) Panoramic view of multiple lungs in the same axial tomographic section.

Fifty animals/group were scanned at 0, 4, 8, 12 or 16^th^ weeks post-inoculation (10 mice/time after inoculation). Mice were anesthetized with ketamin hydrochloride (Park, Davis & Company, Berlin, Germany; 100 mg/kg) and Xylazine (Bayer, Brazil; 10 mg/kg) and placed in prone position inside polypropylene tubes (50 ml), which were arranged together in a wood box with parallel holes (**Figure 1** **A, B**). All the animals were placed with their noses in the same vertical plane. Each animal had a code for future identifications. The box containing the mice was then placed in the CT gantry for thorax scanning (**Figure 1** **C, D**).

CT images were taken in a multislice CT-scanner *Lightspeed*, (General Electric, EU of 16 canals) applying 140 kV with 165 mAs/second (Kernel U90). Thin-section slices, each of 0.625 mm in thickness, and spaced 1 mm apart covered the complete mouse lung from the apex to the hemidiaphragm. Images were acquired in axial plane and the bone algorithm was applied to better visualize the lung. Field of view was 18 cm to include simultaneously all the animals, with matrix of 512×512 and acquisition time of one second per section. Around 20 to 22 slices covered the entire animal lungs.

Following the scan, the mice were placed in their cages, recovering from the anesthesia within 35 minutes. Animals were supplied with standard laboratory diet and water *ad libitum*.

### HRCT-Image Examination

Image analysis was performed independently and blindly by two radiologists from the Radiology Department of the University Hospital San Vicente de Paul (Medellín, Colombia). Images were visualized using *Advantage workstation version 4.3 General Electric*, applying lung and mediastinal windows.

**Figure 2:**
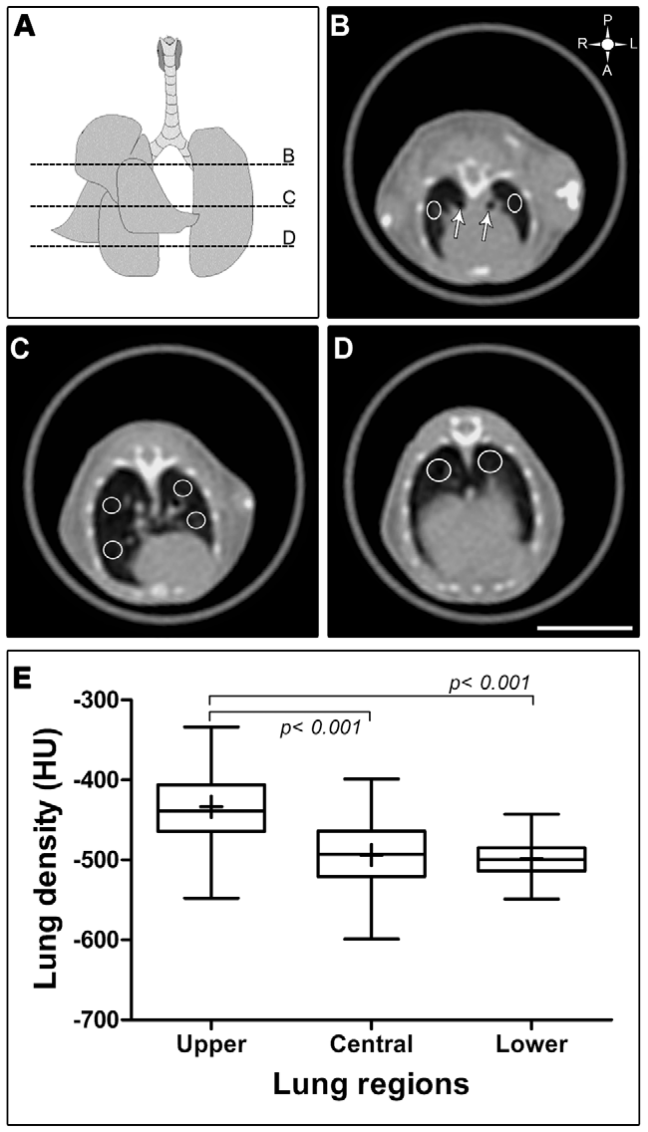
Quantification of lungs density in healthy BALB/c mice. (A) Mouse pulmonary figure in coronal view from a healthy BALB/c mice showing four right lobes and a unique left lobe. Dotted lines correspond to the places where selected tomographic slices were taken: upper or hilar (B), central or middle (C) and lower or lung bases (D) The upper left symbol in figure B, gives the spatial position of posterior or dorsal (P), anterior or ventral (A), left (L) and right (R) regions. (E) Box plot showing the upper, central and lower pulmonary density in healthy BALB/c mice. Density was expressed by Hounsfield units (HU) scale and was measured in ten animals at 7, 11, 15, 19 and 23 weeks of age. One-way ANOVA was used to compare the groups. Upper lung density showed a statistically significant difference in relation to central and lower lung zones, *p*≤*0.001*. ns  =  non significant.

The pulmonary densities were evaluated as described by Plathow (2004) [14], with some modifications. Representative tomographic slides were used to figure out Hounsfield units (HU). Briefly, eight regions of interest (ROI) were selected in the following areas of the right and left lungs: upper or hilar region (about 5 slides below the apex, where the main bronchi enter the lungs), anterior and posterior middle or central region (about 12 slides bellow the apex where the heart presents its larger diameter) and finally, lower lung region (about 18 slides bellow the apex, corresponding to bases of lung). These circles were of 2 mm^2^ for upper and middle regions and of 4 mm^2^ for the lower regions. Main bronchi and vessels were omitted to measure parenchyma density **(** **Figure 2** **, A–D)**. However, to evaluate the extension of the inflammatory infiltrate that presented a predominantly axial location, the ROIs were enlarged to include the interstitium around pulmonary arteries and bronchi.

### Histopathological Analysis

After scanning, ten animals per group were euthanized by Thiopental overdose (Sandoz GmbH. Kundl Austria; 1 ml at 2.5%, i.p) at 0, 4, 8 12 and 16^th^ weeks post-inoculation in accordance to animal ethical practice. Lungs of five mice were intracardially perfused with 10% formalin neutralized with phosphate-buffered saline, removed and fixed in the same solution by at least 48 h. Lungs of remainder mice were used for other studies.

Formalin-fixed lungs were embedded in paraffin and coronal sections (5 µm) were stained with hematoxylin-eosin (HE), and picrosirius with fast green (PIFG) [24] to evaluate the inflammatory reaction and determine collagen deposition, respectively. Slides stained by PFIG were automatically scanned by ScanScope® CS (Aperio, USA). The extent of tissue involvement was estimated by histomorphometry, as described below.

### Histomorphometry of the Lung Inflammatory Area

Morphometric analysis was done using one panoramic image of both lungs per mouse captured with digital camera (Axiocam MRc5, Carl Zeiss, Germany) adapted to a stereomicroscope (Stemi Sv11, Carl Zeiss, Germany). The images were analyzed by the free Image J software (http://rsbweb.nih.gov/ij/, NIH, USA). Areas of interest (AOIs), correspondent to the inflammatory regions, were manually drawn and measured. The percentage of pulmonary area with inflammatory reaction was calculated dividing the sum of total AOIs by the total area occupied with lung tissue (excluding the air space).

### Statistical Analysis

The statistical analyses between groups were performed with Prism 5.0 software (Graph Pad, USA) applying one-way or two-way ANOVA. Values were expressed as mean ± standard error of the mean. *p values* less than *0.05* were considered statistically significant; *p values* less than *0.01* were considered statistically highly significant. The frequency of infected mice with increased lung density was determined considering the outlier values established by boxplot graph, using 1.5 times of the interquartile range.

## Results

### HRCT in Healthy Mice

Lung density in healthy BALB/c mice showed local differences according to the region evaluated. The upper or hilar lung density was higher (−432.7±10.82) than the central and lower regions, −492.4±6.5 and −492.7±4.8, respectively, during all observation times, indicating that these latter regions were more aerated than the former; *p*≤*0.001,* **(** **Figure 2E** **)**.

### HRCT in *P. brasiliensis* Infected Mice

**Figure 3:**
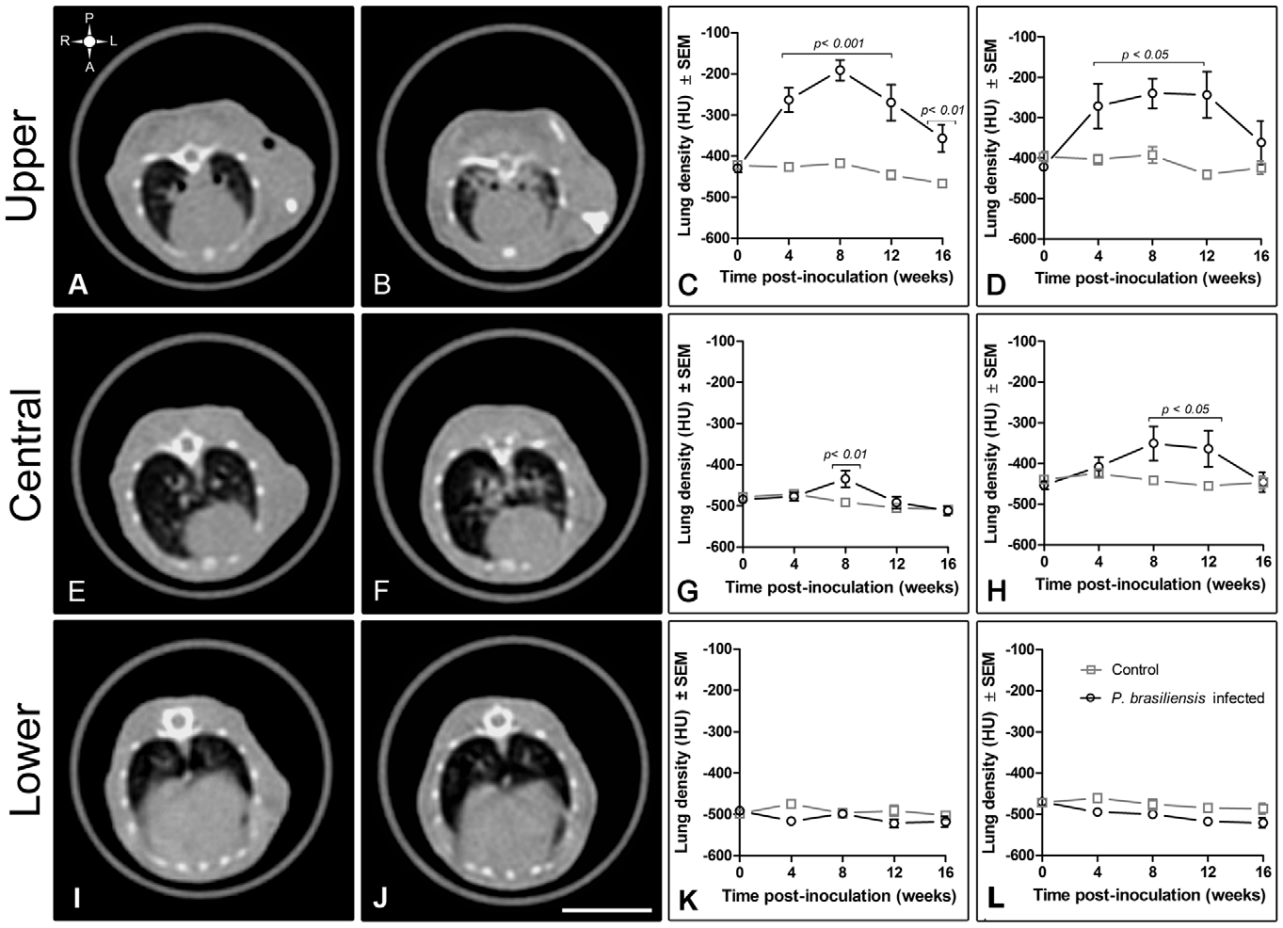
Topographic location of the main pulmonary lesions and sequential changes of the lungs density in the experimental PCM model. Representative HRCT images of a lung control (A, E, I) and *P. brasiliensis* infected mice (B, F, J) at 12 weeks of follow-up. The upper left symbol in figure A, gives the spatial position of posterior or dorsal (P), anterior or ventral (A), left (L) and right (R) regions. Tomographic slices, selected as described in figure 1, represent upper (A, B, C, D) central (E, F, G, H) and lower (I, J, K, L) pulmonary regions. Peri-bronchial and bilateral consolidations around pulmonary hilum in upper (B) and central lung regions (F) of a *P. brasiliensis* infected mice. Quantification of lung density in infected animals (C, D, G, H, K, L) was assessed selecting pulmonary parenchyma, excluding main vessels and bronchi (C, G, K), or including the hilum (D, H, L). Measures are expressed as Hounsfield units (HU) ± SEM and were acquired at 0, 4, 8, 12 and 16 weeks pos-inoculation. Gray squares represent control group and black circles, *P. brasiliensis* infected group. Two-way ANOVA was used to compare groups. Scale bar in tomographic images  = 1 cm.

At week 4 post-infection, *Pb*-infected mice showed peri-bronchial consolidations that persisted in every one of the evaluation periods. Pulmonary consolidations was associated mainly with a significant increase in upper lung density as compared with controls, −263±29 vs. −426±8 HU at week 4 (*p<0.001*), −191±25 vs. −403±17 HU, at week 8 (*p<0.001),* −269±43 vs. −445±12, at week 12 (*p<0.001).* At week 16, upper consolidations tended to decrease as well as the corresponding density, −356±33 vs. −466±9 at week 16 (*p>0.01*) **(** **Figure 3C** **)**. At weeks 8 and 12, consolidation had progressed to involve also the middle regions with a statistically significant increase in density **(** **Figure 3G, H** **)**. The lesions, during every one of the infection times, were mainly located in the hilar region **(** **Figure 3D, H** **)**. Lesions were not detected in the lung bases, which presented normal density **(** **Figure 3** **I-L)**.

**Figure 4:**
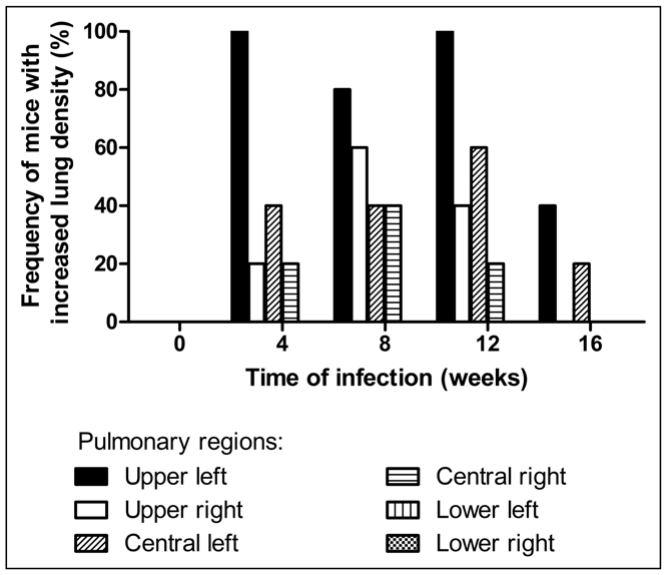
Frequency of increased lung density according to different lung regions and time of *Pb*-infection. The infected mice with increased lung density were selected using the upper limit value of density established in normal animals. Note upper left lung predominance of lesions (black bar) in the majority of mice during all evaluation times.

Left lung was more frequently affected by lesions than the other pulmonary regions (>80% the mice at week 4, 8 and 12), followed, in frequency, by upper right and central or middle lung regions, saving the bases (**Figure 4** **)**.

**Figure 5:**
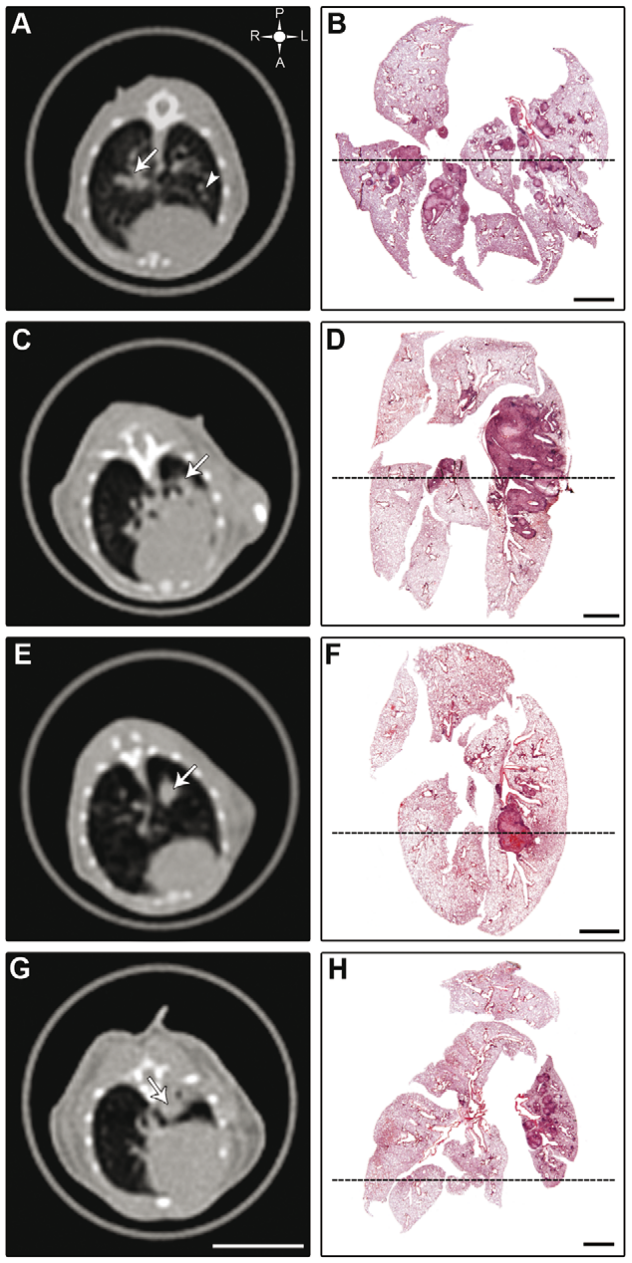
Comparison of the main lung lesion patterns accessed by HRCT and histopathology in the experimental model of PCM. Figures A, C, E, G correspond to HRCT images and B, D, F, H show the corresponding histopathological lesions taking in coronal plane. The upper left symbol in (A) indicates posterior or dorsal (P), anterior or ventral (A), left (L) and right (R) regions. Dotted lines show an approximated position of the tomographic section. (A) Large nodular lesion represented by a peri-bronquial consolidation (arrow) is located at right hilar region and other left small nodules are indicated by arrowhead. (B) Several nodules with varied sizes. Only the larger nodules are easily seen by tomography. (C) Confluent lesion expressed by left central peri-bronchial consolidation (arrow) extended from the hilum to large area of the parenchyma. (D) Consolidated areas of perivascular and justabronchial granulomatous lesions. (E) Pseudotumoral lesion defining a left central pulmonary mass (arrow). (F) Left central pseudotumoral mass obstructing the bronchus. (G) Left lung with accentuated atelectasis (arrow). (H) Lung section corresponding to figure G showing inflammatory periarterial nodules tending to confluence. Scale bar for HRCT images  = 1 cm. Scale bar for histopathological images  = 2 mm.

The main patterns of lesions were nodular-diffuse, confluent and pseudo-tumoral with some occasional and additional aspects such as atelectasis. These aspects were revealed by both HRCT and histopathology, showing a strong correspondence between the two approaches (**Figure 5**).

**Figure 6:**
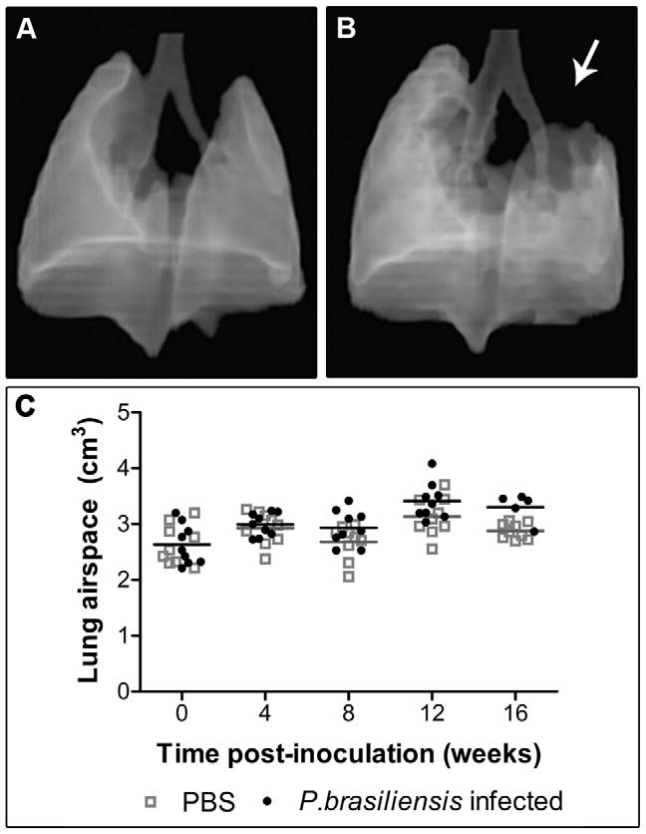
Tomographic reconstruction of pulmonary air space and its quantification in the experimental model of PCM. Three-dimensional aspect of lungs from control (A) and *P.b* infected mice (B), showing a none insufflated area in the upper left lung (arrow in B) and compensated hyper-insufflation in the opposite lung. Scatter dot-plot of air space volume of lung (cm^3^) (C) measured at 0, 4, 8, 12 or 16 weeks post inoculation with PBS (gray squares) or *P. brasiliensis* conidia (black circle). There was no significant difference between the groups at anytime during the experimental process.

The three-dimensional reconstruction of air-structure showed that the more consolidated zones, predominantly in the left upper lung, provoked a deprivation of the air volume in the correspondent region **(** **Figure 6B** **arrow)**. Nonetheless, the quantification of the total lung volume did not show statistically significant difference between control and infected animals at any time of evaluation (**Figure 6C** **).**

The cardio-thoracic ratio, vascular structures and main bronchi did not show significant changes at any time during evaluation and no mouse showed a radiologic pattern compatible with lung fibrosis, a common sequela observed in human PCM. Pleural changes were observed in only one mouse.

### Histopathology Analysis

The granulomatous reaction was initially seen around bronchi, bronchioli and blood vessels, where various compact nodule-shaped granulomas were detected **(** **Figure 5B** **).** Some of them became confluent and contained numerous fungal cells. Additionally, large areas of the lung became consolidated involving hilar regions and lung parenchyma **(** **Figure 5D** **).** After 12^th^ week of infection, confluent granulomas formed pseudotumoral masses **(** **Figure 5F** **)** that obstructed large bronchi. Complete atelectasis of the left lung was observed in one animal **(** **Figure 5H** **)**.

**Figure 7:**
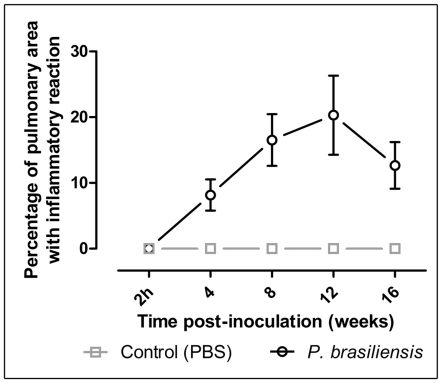
Percentage of the lung area with inflammatory reaction in *P. brasiliensis* infected mice at different times of infection. The lung area with inflammation was assessed by histomorphometry as described in M&M. Five control (gray) and *Pb*-infected (black) mice were evaluated at 2h, 4, 8, 12 and 16 weeks after inoculation.

**Figure 8:**
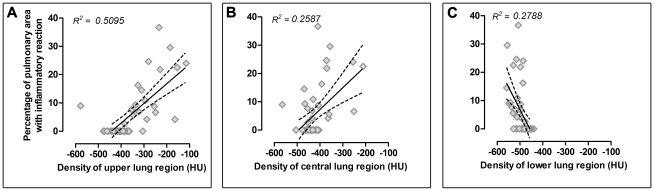
Correlation between percentage of pulmonary area with inflammatory reaction and lung density. Pulmonary inflammation measured by histomorphometry was correlated with HUs obtained in the upper (A), central (B) and lower (C) regions. Both parameters were directly correlated when the upper and the central lung density were used (*r^2^ = 0.5095*, *p<0.0001* and *r^2^ = 0.2578*, *p<0.0002,* respectively); and inversely correlated with the lower lung density (*r^2^ = 0.2788*, *p<0.0001)*.

The percentage of lung area occupied by inflammatory reaction was 8±2% at 4^th^ week, and gradually increased to 20±6% at 12^th^ week. Finally, at 16^th^ week, the percentage of affected lung area decreased to 13±3% (*p<0.01* at all evaluation times) **(** **Figure 7** **)**. The extension of the lesions presented a direct correlation with lung density in the upper **(** **Figure 8A** **)** and central region **(** **Figure 8B** **)**, while an inverse correlation was observed in the lower lung region **(** **Figure 8C** **)**.

By histopathology, the presence of fibrosis was ascertained; it was observed predominantly in the inflammatory reaction around arteries and in the periphery of granulomas, with less intensity in the peribronchial connective tissue and without evidence in the interstitium.

## Discussion

Through a comparative analysis between HRCT and histopathological data, this work revealed that noninvasive conventional medical X-ray tomography is adequate to follow the sequential lung lesions in experimental PCM in mice. This procedure allowed detection of the main pathological patterns, the differential topographic distribution of the pulmonary lesions in both lungs, and their intensity in our experimental model of PCM. Three basic lesion patterns were evidenced by the study: nodular-diffuse, confluent and pseudo-tumoral **(** **Figure 5** **).** Histopathologically, the lesions were predominantly of the granulomatous type and were mainly located around branches of the arterial vasculature and close to the bronchial tree, preserving large areas of the parenchyma. Concerning the topographic distribution, the hilar region of the upper left lung was more frequently involved than other regions **(** **Figures 3** **and** **4** **)**, and while the mechanism of this preference is unknown, it could be influenced by lymphatic drainage. It was surprising to notice the predominance of left lung involvement, considering that the bronchial mouse anatomy reveals a thinner main left bronchus in comparison with the right one and with approximately the same angular deviation from the carina **(** **Figure 6** **)**. We do not know the effect of the ventral decubitus position on the differential inclination of the main bronchi.

The intensity of the inflammatory reaction, evaluated by histomorphometry, was crescent until the 12^th^ week of infection with subsequent decrease due to the tendency to form predominantly compact and more isolated pseudotumoral masses **(** **Figure 7** **)**. This histopahological behavior was also detected by HRCT, as expressed by lung density measures **(** **Figure 3** **)** that showed a significant correlation mainly in the upper or hilar lung region **(** **Figure 8A** **)**.

The absence of difference in the total lung air space volume between the control and infected groups **(** **Figure 6** **)** suggested that the healthy or less affected lung compensated, by hyper-insufflation, the focal volume lost. This effect was also supported by the indirect correlation observed between the percentage of lung area with inflammatory reaction and the lung density observed in the lower region **(** **Figure 8C** **)**.

For future studies, it would be of interest to assess pulmonary function in our experimental model of PCM by non-invasive methods such as unrestrained whole-body plethysmography for small animals, or other more accurate procedures for determining physiological parameters although invasive techniques, such as forced pulmonary maneuvers system or forced oscillation techniques [25]. Pulmonary function analysis would respond to the following question: Do the large masses or the consolidated lesions observed in our study by HRCT or the fibrosis recorded by histopathology, decrease the normal function of lungs of mice with experimental PCM?

Although the human and mouse lungs exhibit basic anatomic similarities, they present significant interspecies differences, such as: the absence of respiratory bronchioles in the mouse; number of the subdivisions of the conducting airways; characteristics of pleura structure, interlobular septa, pulmonary and bronchial vasculature, bronchial associated lymphoid-tissue and others that could contribute to explain the different behaviours of fibrogenesis [26], [27], [28].

Radiologic patterns showed by HRCT in the experimental infection, differed slightly from their counterpart in human patients. The most frequent HRCT findings in patients with pulmonary PCM are: ground-glass attenuation areas, small centrilobular, cavitated and large nodules, parenchymal bands, airspace consolidations, interlobular septal thickening, architectural distortion, traction bronchiectasis, paracicatritial emphysema and fibrosis. Most of those HRCT findings predominate in the periphery and posterior regions involving all lung zones, with light predominance in the middle zones [29], [30], [31], [32]. The radiologic patterns described above in patients with pulmonary PCM were dependent on the stages of the disease and the exclusion or not of patients who had received previous treatment.

Values of lung density of healthy mice differed from those of the human counterpart. In BALB/c mice the apices were denser than the bases suggesting that there was less air in the former regions **(** **Figure 2** **)**. On the contrary, human lung apices are more ventilated and less perfused than bases which suppose a decrescent button to up gradient of density probably due to human upright position, which considerably reduced the flow of low pressure pulmonary artery blood in the upper lung for long periods. Otherwise, ventilation to the various parts of the lung is much less affected by body position. As a consequence, oxygen uptake and carbon dioxide excretion are impaired in the upper as compared with the lower parts of the lungs in the erect position. This situation produces a more oxygenated environment in the upper lungs, which favor proliferation of some pathogenic agents like *Mycobacterium tuberculosis* [33]. This kind of mechanism appears not to interfere on paracoccidioidomycosis infection in human and experimental animals.

In conclusion, this study demonstrated for the first time that conventional-HRCT is a useful, precise and non-invasive technique to evaluate and quantify the pulmonary damage occurring in the mouse experimental paracoccidioidomycosis. This procedure will contribute significantly to decreases the need of killing large number of animals, and to monitor treatment efficacy in animal models with an approach that reflecting the way human pulmonary diseases are studied.

## Supporting Information

Alternative Language Abstract S1 Translation of the Abstract into Spanish by author Angela Restrepo.
(0.03 MB DOC)

Alternative Language Abstract S2 Translation of the Abstract into Portuguese by author Luz Elena Cano.
(0.03 MB DOC)
