# Supplementary material for: Pulmonary Abnormalities in Mice with Paracoccidioidomycosis: A Sequential Study Comparing High Resolution Computed Tomography and Pathologic Findings
Source: PLoS Negl Trop Dis. 2010 Jun 29;4(6):e726. doi: 10.1371/journal.pntd.0000726 (PMC2894136; doi:10.1371/journal.pntd.0000726)
Supplement: Alternative Language Abstract S1 — Translation of the Abstract into Spanish by author Angela Restrepo. (0.03 MB DOC) [file pntd.0000726.s001.doc]

Translation of the abstract “Pulmonary Abnormalities in Mice with Paracoccidioidomycosis: A Sequential Study Comparing High Resolution Computed Tomography and Pathologic findings” into language Spanish by author Angela Restrepo

La paracoccidioidomicosis humana (PCM) es una micosis sistémica endémica de origen pulmonar. Hasta el presente, no se conocen estudios de imaginología que midan el desarrollo de las lesiones pulmonares resultantes de la infección experimental en la PCM. Este estudio pretende definir en ratones con PCM infectados experimentalmente los patrones, la topografía y la intensidad de las lesiones pulmonares, mediante un análisis comparativo entre tomografía axial computada de alta resolución (TACAR) y parámetros histopatológicos.

**Metodología.** Se inocularon intranasalmente ratones BALB/c machos con 3x10^6^ conidias de *Paracoccidioides brasiliensis* (*Pb*) (*n=50*) o PBS (*n=50*). Se realizó la TACAR cada cuatro semanas para definir las lesiones pulmonares, cuantificar la densidad tisular, reconstruir y cuantificar la estructura aérea del pulmón. Los pulmones se analizaron posteriormente por histopatología e histomorfometría.

**Resultados.** Los métodos anteriores permitieron evidenciar tres patrones diferentes de lesiones: Nodular difuso, confluente y pseudotumoral. Estas lesiones se localizaban principalmente alrededor del hilio y comprometían con más frecuencia el pulmón izquierdo. A la cuarta semana post-infección, se evidenciaba mediante TACAR que el 80% de los ratones infectados con *Pb* presentaban consolidaciones peribronquiales en el tercio superior del pulmón, asociadas a un incremento significativo de la densidad pulmonar con respecto al grupo control, (-263±25 vs. -422±10 HU, *p<0.001*). A la octava y decimosegunda semana post-infección, las lesiones se extendieron hasta comprometer la región media del pulmón.

El análisis histopatológico estableció que las consolidaciones observadas mediante TACAR correspondían histológicamente a una reacción granulomatosa confluente, mientras que los nódulos coincidían con granulomas individuales, compactos y bien definidos. A la decimosexta semana post-infección, los granulomas confluentes formaban masas pseudotumorales que obstruían grandes bronquios. Se observó que gradualmente aparecía fibrosis focal alrededor de los granulomas pero esta secuela solo se evidenció por histopatología y no por TACAR.

**Conclusiones y relevancia.** Este estudio demostró que la TACAR es una herramienta útil para la evaluación y cuantificación de las lesiones pulmonares observadas en la PCM experimental en el ratón. El diseño experimental utilizado evita la necesidad de sacrificar un número elevado de animales y podría servir para evaluar la eficacia de un tratamiento por técnicas que se aproximan más a las empleadas en el estudio de enfermedades respiratorias humanas.
