# Supplementary material for: Pulmonary Abnormalities in Mice with Paracoccidioidomycosis: A Sequential Study Comparing High Resolution Computed Tomography and Pathologic Findings
Source: PLoS Negl Trop Dis. 2010 Jun 29;4(6):e726. doi: 10.1371/journal.pntd.0000726 (PMC2894136; doi:10.1371/journal.pntd.0000726)
Supplement: Alternative Language Abstract S2 — Translation of the Abstract into Portuguese by author Luz Elena Cano. (0.03 MB DOC) [file pntd.0000726.s002.doc]

Translation of the abstract “Pulmonary Abnormalities in Mice with Paracoccidioidomycosis: A Sequential Study Comparing High Resolution Computed Tomography and Pathologic findings” into language Portuguese by author Luz Elena Cano.

**Fundamentos:** A Paracoccidioidomicose humana (PCM) é uma doença fúngica endêmica de origem pulmonar. A monitoração das lesões pulmonares através de estudos de imagem num modelo experimental da PCM não tenha sido previamente tentada. Estes focos de estudo sobre a definição de padrões, topografia e intensidade das lesões pulmonares presentes nos camundongos infectados experimentalmente com conídios de P.brasiliensis, foi feita por meio de uma análise comparativa entre tomografia computadorizada de alta resolução (TCAR) e os parâmetros histopatológicos.

**Metodologia:** Camundongos BALB/c do sexo masculino foram inoculados pela via intranasal com 3x10^6^ conídios de P. brasiliensis (Pb) (n = 50) ou PBS (n = 50). TCAR foi realizada a cada quarta semana para determinar lesões pulmonares, quantificar a densidade pulmonar, reconstruir e quantificar a estrutura de ar do pulmão. Os pulmões também foram analisados por histopatologia e histomorfometria.

**Resultados:** Três diferentes padrões de lesões pulmonares foram evidenciados pela TCAR e histopatologia, como segue: nodular difuso, confluente e pseudo-tumoral. As lesões foram localizadas principalmente ao redor do hilo e mais freqüentemente observadas no pulmão esquerdo. Na quarta semana da infecção, a TCAR mostrou que 80% dos camundongos infectados com o fungo tinha consolidações peri-brônquica associadas com um aumento significativo na densidade pulmonar superior quando comparados com os animais controles, (-263 ± 25 vs -422 ± 10 HU, p <0,001). Após das 8 e 12 semanas da infecção, a consolidação progrediu envolvendo também as regiões média. Ar-estrutura mostrou que a reconstrução das zonas mais consolidadas provocou uma privação do volume do ar na região correspondente. O exame histopatológico revelou que a consolidação, avaliada por TCAR, foi equivalente histologicamente a uma reação granulomatosa confluente, enquanto nódulos corresponderam aos granulomas individuais compactos. Após da semana 12 da infecção, granulomas confluentes, massas pseudotumorais que obstruíam os brônquios de grande porte foram observados. Discreta fibrose focal era visível gradualmente em torno dos granulomas, mas esta constatação foi evidente apenas pela histopatologia.

**Conclusões/ Significado:** Este estudo demonstrou que a TCAR convencional é uma ferramenta útil pra a avaliação e quantificação dos danos pulmonares que ocorrem na paracoccidioidomicose experimental nos camundongos BALB/c. O delineamento experimental utilizado diminui a necessidade de sacrificar um grande número de animais, e serve para monitorar a eficácia do tratamento por meio de uma abordagem mais racional para o estudo desta doença do pulmão humano.
